# The Implementation of Internet Interventions for Depression: A Scoping Review

**DOI:** 10.2196/jmir.5670

**Published:** 2016-09-08

**Authors:** Filip Drozd, Linda Vaskinn, Hans Bugge Bergsund, Silje Marie Haga, Kari Slinning, Cato Alexander Bjørkli

**Affiliations:** ^1^ National Network for Infant Mental Health Centre for Child and Adolescent Mental Health, Eastern and Southern Norway Oslo Norway; ^2^ Centre for Child and Adolescent Mental Health, Eastern and Southern Norway Oslo Norway; ^3^ Department of Psychology University of Oslo Oslo Norway

**Keywords:** depression, scoping review, implementation, Internet interventions

## Abstract

**Background:**

Depression is one of the most common mental health problems among adults, but effective treatments are not widely accessible. The Internet holds promise as a cost-effective and convenient delivery platform of interventions for depression. However, studies suggest that Internet interventions are not widely available in routine settings.

**Objective:**

The aim of this study was to review the literature and examine whether there are systematic differences in reporting of the various implementation components on Internet interventions for depression, and then to examine what is known about and is characteristic of the implementation of these Internet interventions in regular care settings.

**Methods:**

We performed a scoping review, drawing upon a broad range of the literature on Internet interventions for depression in regular care, and used the active implementation framework to extract data.

**Results:**

Overall, the results suggested that knowledge about the implementation of Internet interventions for depression in regular care is limited. However, guided support from health professionals emphasizing program adherence and recruitment of end users to the interventions emerged as 2 main themes. We identified 3 additional themes among practitioners, including their qualifications, training, and supervision, but these were scarcely described in the literature. The competency drivers (ie, staff and user selection, training, and supervision) have received the most attention, while little attention has been given to organizational (ie, decision support, administration, and system intervention) and leadership drivers.

**Conclusions:**

Research has placed little emphasis on reporting on the implementation of interventions in practice. Leadership and organizational drivers, in particular, have been largely neglected. The results of this scoping review have implications for future research and efforts to successfully implement Internet interventions for depression in regular care.

## Introduction

According to the World Health Organization [1,2], about 350 million people have depression worldwide every year. However, less than 50% (in some countries, less than 10%) of those affected have access to or seek professional help [3]. Barriers to treatment include limited access to effective treatments, stigma, undertreatment, and lack of trained providers [4]. Internet interventions have been proposed as one innovate way to overcome such barriers, and systematic reviews show that people can benefit from both unguided and therapist-supported interventions [5-8]. Despite these findings, Internet interventions are not commonly used in practice, and their uptake and actual reach, among both practitioners and users, appears to be low (eg, see [9-11]). It is, therefore, necessary to understand factors that can explain the gap between our knowledge about the effects of Internet interventions and how to translate these findings into practice.

### Barriers to Uptake of Internet Interventions

A lack of availability of Internet interventions and, thus, experience with such a treatment modality has been identified as one important barrier to their uptake and use in practice [12]. Thus, it makes sense that a lack of training in the delivery of Internet interventions is another barrier [9,13]. However, recent studies showed that discrete implementation strategies, such as availability or training, would not necessarily translate an innovation into sustainable changes in practice. In a study by Friesen et al [14], 12 graduate students completed a training workshop in the delivery of Internet-based cognitive behavioral therapy (iCBT) for depression, anxiety, and panic disorder; 1 year after the workshop, each student had treated, on average, only 3 clients. In another study, conducted by Wilhelmsen and colleagues [15], only 1 of 11 general practitioners deployed the guided MoodGYM program as prescribed after training, even though the general practitioners had expressed a desire to acquire iCBT as a tool in their treatment of depression. Thus, more multifaceted strategies for implementing Internet interventions seem necessary, especially for more complex interventions and large-scale implementation.

Earlier experiences from the Improving Access to Psychological Therapies program in the United Kingdom showed that iCBT can be implemented effectively using various configurations and setups (ie, implementation strategies; [16]). However, there were insufficient data to clearly demonstrate that any one configuration was superior to another, which necessitated setups based on the needs of the local population and services (ie, bottom-up implementation). A more recent examination of iCBT in primary care trusts across England [17] identified more-specific barriers to national implementation: (1) availability of alternative interventions, (2) supporter attitudes, and (3) organizational issues such as management support, funding, and intraorganizational communication. These findings allow for a more top-down implementation and emphasize the need for multifaceted, multilevel approaches to implementation (eg, see [18]); that is, a need to deploy several implementation strategies across different levels in a health care service, to ensure successful integration of an intervention in practice. This also means having models of service delivery that describe the practical implementation of Internet interventions as a part of health care services.

Service delivery models would not only describe the practical implementation of Internet interventions, but also provide the infrastructural, legal, managerial, and institutional frameworks needed to operate and maintain Internet interventions as a service. However, service delivery models have not been adequately described in the literature [19-22], although variations of stepped-care models have been proposed (eg, see eg [23]). In stepped care, Internet interventions are suggested as a first step, while reserving more intensive and resource-demanding treatment for those who do not respond and for the most severe cases. One study in the United Kingdom found that the implementation of computer-based cognitive behavioral therapy as stepped care within a specialist service actually increased service capacity by approximately 50% [24].

There are also alternatives to stepped care, such as the centralized unit model [25]. The centralized unit, which is responsible for the Web app, training and supervision of therapists, and screening and referral of patients to practitioners, is considered to be a cost-efficient model, providing a high degree of oversight and quality control. A similar model has been used in Sweden and has helped achieve desirable results [26], albeit in a small number of clinics. However, a centralized unit model with a high degree of control may not be viable for large-scale implementation, where a decreasing degree of control and more variability in performance may be expected (eg, see [27]). Thus, more work is needed to enable integration and dissemination of Internet interventions in practice. In this regard, a first step is to map the state-of-the-art of the implementation of Internet interventions in routine practice and to identify any knowledge gaps in the literature.

### Aims of This Study

The overall aim of this study was to review what is known about the implementation of Internet interventions for depression in regular care, based on the scientific literature. More specifically, the goal was, first, to examine whether there are any systematic differences in the reporting of different aspects of the implementation of Internet interventions, and thereby identify any gaps in the literature. Second was to examine what characterizes the literature on implementation of Internet interventions for depression in terms of core implementation components.

## Methods

### Study Design and Search Strategy

We conducted a scoping review, which has the purpose of identifying gaps in the literature by systematically assessing the breadth of a body of literature in a particular area, rather than the narrow and specific research questions typical of systematic reviews such as meta-analyses [28,29]. The search was conducted by a medical librarian, using the following scientific databases: (1) ISRCTN registry, (2) OpenGrey, (3) Ovid MEDLINE, (4) PsycINFO, (5) PubMed, (6) Web of Science, (7) World Health Organization International Clinical Trials Registry Platform, (8) CINAHL, (9) ClinicalTrials.gov, (10) Cochrane, (11) Embase, and (12) Google Scholar. Google Scholar was only used for additional searches, as it is not a traditional scientific database and has been found unsuitable for systematic literature search [30].

Search terms consisted of the combination of (1) internet, (2) intervention, and (3) depression, including synonyms for all terms (for the complete search strategy, see Multimedia Appendix 1). The inclusion of the term implementation and its synonyms (eg, adoption, integration, and dissemination) often used in the literature produced a large and unmanageable number of irrelevant search results initially (ie, 35,000–45,000 articles per database) due to its inconsistent use and different definitions in various disciplines (also, see the Study Selection subsection below). Thus, it was not feasible to include implementation in the search strategy. The final search included references published between 1946 and March 24, 2014. After running an initial duplicate check, we imported the search results to Mendeley Desktop v1.13.8 (Mendeley Ltd). After the initial screening process, we also hand searched reference lists in identified reviews and meta-analyses, as well as relevant journals (for a list of journals, see Multimedia Appendix 2). We also contacted researchers involved in the European and international societies for research on Internet interventions [31,32].

### Study Selection

Raters (HBB and LV) independently reviewed all references for eligibility based on their title, abstract, and author-provided keywords. Included references had to study (1) an Internet-based (2) intervention for (3) depression in (4) a regular care setting or (5) clearly indicate examining concepts relevant for implementation (eg, dissemination, fidelity, acceptability, and effectiveness). Systematic reviews and nonempirical references such as trial protocols, book reviews, editorials, magazine articles, and theoretical or methodological articles were excluded. Studies clearly identified as efficacy trials and offline interventions, such as desktop-based, computer-based, and CD-ROM interventions, were also excluded from this review. Efficacy trials are conducted in highly controlled settings and outside of regular care, and we did not expected them to contribute to our research questions. We included only references in English and Scandinavian languages in the coding process. In case of disagreements between the 2 reviewers, agreement was reached through discussion. Agreement between the 2 coders was estimated using Cohen kappa, resulting in a coefficient of 0.72 (95% CI 0.64–0.81), which is considered to be good [33].

### Implementation Components

In order to systematically extract data, we applied the active implementation framework (AIF) developed by Fixsen et al [34]. In their comprehensive review, they identified a set of core implementation components, which they described as “the most essential and indispensable components of an implementation practice or program” [34]. These are (1) staff and client selection, (2) training, (3) supervision, (4) performance assessment, (5) decision support, (6) administrative support, (7) system intervention, and (8) leadership. These core components are considered universal and apply to all efforts of implementing an intervention in practice. However, they are also considered compensatory, such that weaknesses in one of the components may be overcome by the strengths in other components (eg, high-quality coaching and performance assessments may compensate for poor training). Thus, it is not the applied number of components (ie, the more, the better) that determines the quality of implementation, but rather the quality of how these components are carried out. We coded all references as either containing information (1 = yes) on the respective implementation components or not (0 = no). For each reference coded on an implementation component, we extracted corresponding information for the qualitative synthesis.

According to the AIF, selection, training, supervision, and performance assessments (ie, treatment fidelity) are referred to as the *competency drivers* [35]. These are concerned with the development, improvement, and sustainment of, most often, the practitioners and supervisors’ abilities to work with an intervention in a competent manner. Implementation requires essentially a behavior change by means of training and coaching carefully selected staff in the initial stages of implementation whose performance is assessed (eg, how well practitioners work with the intervention). The context of an intervention also includes a clear definition of the population for whom the program is intended, and the application of inclusion and exclusion criteria to provide safer and better health services to end users. Thus, the competency drivers in this study may also pertain to selection, training, supervision, and assessment of end users.

Decision support, administrative support, and system intervention are the *organizational drivers* [35]. Organizational drivers are concerned with the planning and establishment of support systems, such that new interventions can be implemented effectively. This entails collecting data for continuous quality assurance and improvement (ie, decision support); reducing obstacles by establishing or making changes to internal policies, rules, procedures, routines, organizational culture, and climate (ie, administrative support); and developing strategies to cooperate with external systems to assure the availability of the financial, organizational, and human resources required to support and continue the intervention (ie, system intervention). Finally, the *leadership driver* is the final core component that is important in terms of setting priorities, establishing consensus, offering incentives, and managing the overall process of implementation [36].

We also coded the extracted analysis units on different organizational levels to account for multilevel approaches to implementation, based on the individual, group, leadership, and organizational levels (IGLO) framework [37]. To differentiate between individuals at the receiving end of the intervention and individuals delivering the intervention or providing supervision, we subdivided the individual level into users, practitioners, and supervisors. Certain components and organizational levels seemingly overlap (eg, supervision and leadership). However, adding the IGLO framework to the coding contributes to specifying whether the devised strategies are targeted at formal organizational structures, management, groups, or individuals, and who is the target of those strategies. So including the IGLO framework may help to distinguish units pertaining to, for example, the supervision component that describe the scope of supervision for *supervisors* and units describing who is being supervised (eg, practitioners).

### Data Analysis

We used descriptive statistics to summarize the included studies. To examine whether there were any systematic differences in the reporting between implementation components, we used Cochran Q tests to account for pairwise data (ie, the same or dependent references), while we analyzed qualitative data according to the template approach to examine what characterizes the implementation of Internet interventions in the literature [38].

A template analysis allows researchers to identify a priori themes, which are subsequently revised by theoretical concepts or perspectives that emerge during the analysis and, hence, inform the research question(s). The analysis in our study was consistent with template analysis described by King [39]: (1) defining a priori themes (ie, AIF), (2) extracting analysis units (by LV and HBB), (3) coding on a relevant theme, modifying an existing theme, or devising a new theme (LV and HBB), (4) producing the initial template (LV and FD), (5) developing the template (LV and FD), and (6) interpreting the final template (FD). Finally, each time we modified the template, we reanalyzed preceding units according to the modified template and, in order for a theme to be included in the final template, each theme had to include information from a minimum of 10 references. There are no formal procedures for determining the amount of information necessary to constitute a theme. However, it is generally important to avoid producing very narrow thematic structures and becoming too concerned with fine distinctions at lower levels of the coding hierarchy, which may not help to make sense of the data or re-present data in a disproportionate way. There is no perfect, final template, but a law of diminishing returns applies, and theme saturation is reached when continuing (re-)coding does not enrich the data. For each identified theme, we report the frequencies and percentages, and provide definitions and examples of the themes.

## Results

**Figure 1:**
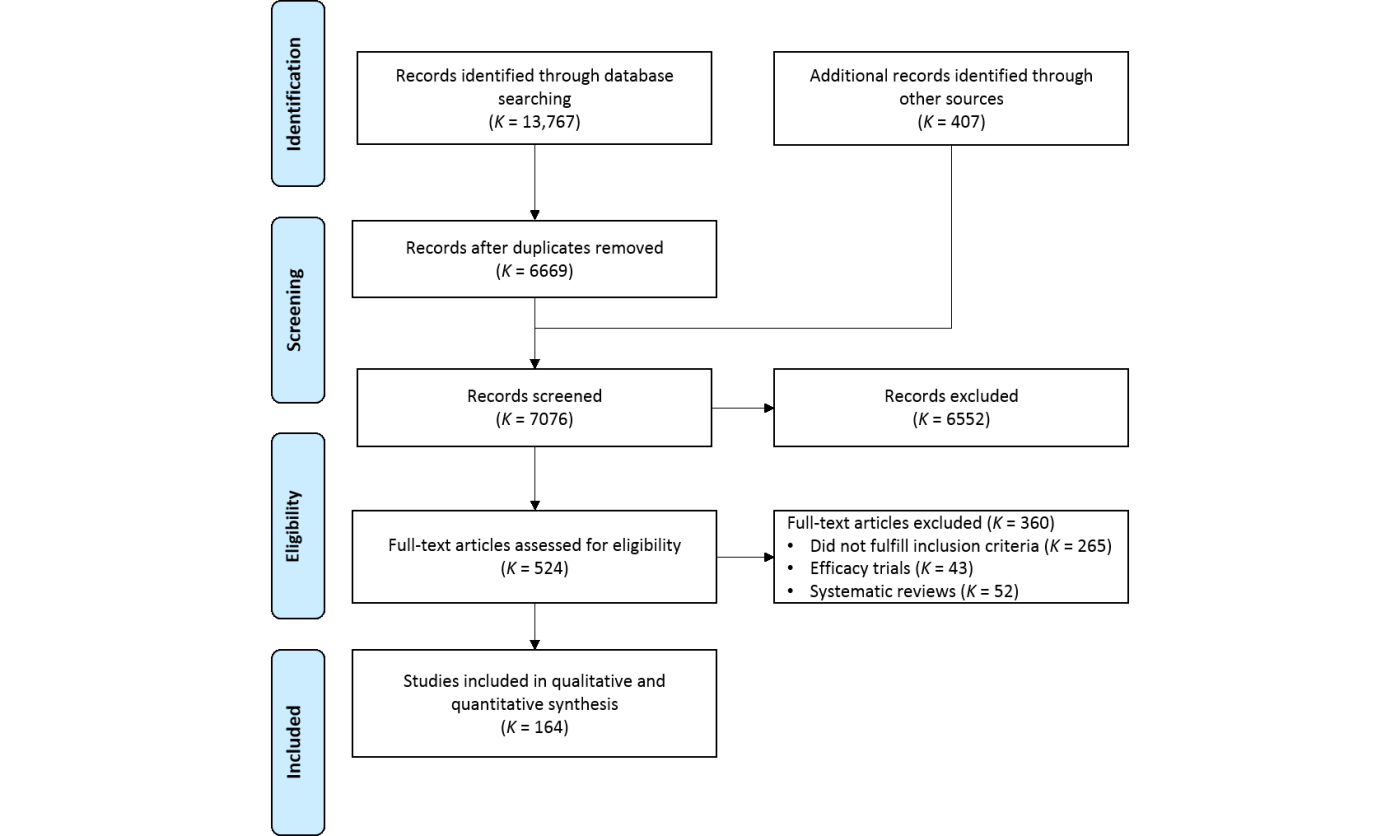
Flow diagram of selection of studies on Internet interventions for depression in regular care settings, 2002 to March 24, 2014.

As Figure 1 shows, the final list included 164 publications (for a complete list, see Multimedia Appendix 3). The main reason for excluding full-text articles was that they did not meet our inclusion criteria (ie, effectiveness or implementation study of an Internet intervention for depression in a regular setting). We also excluded 43 (11.9%) efficacy trials because they were conducted in a university clinic or laboratory or a research context. Hence, the results would have been explainable by the aim of these studies, since these were focused on assessing the effectiveness of interventions in a research context and would not contribute to identifying relevant information about implementation. In addition, we excluded full-text articles on the basis that they were theoretical or methodological articles (*K*=57, 15.8%) or unavailable in English or any of the Scandinavian languages (*K*=28, 7.8%).

**Figure 2:**
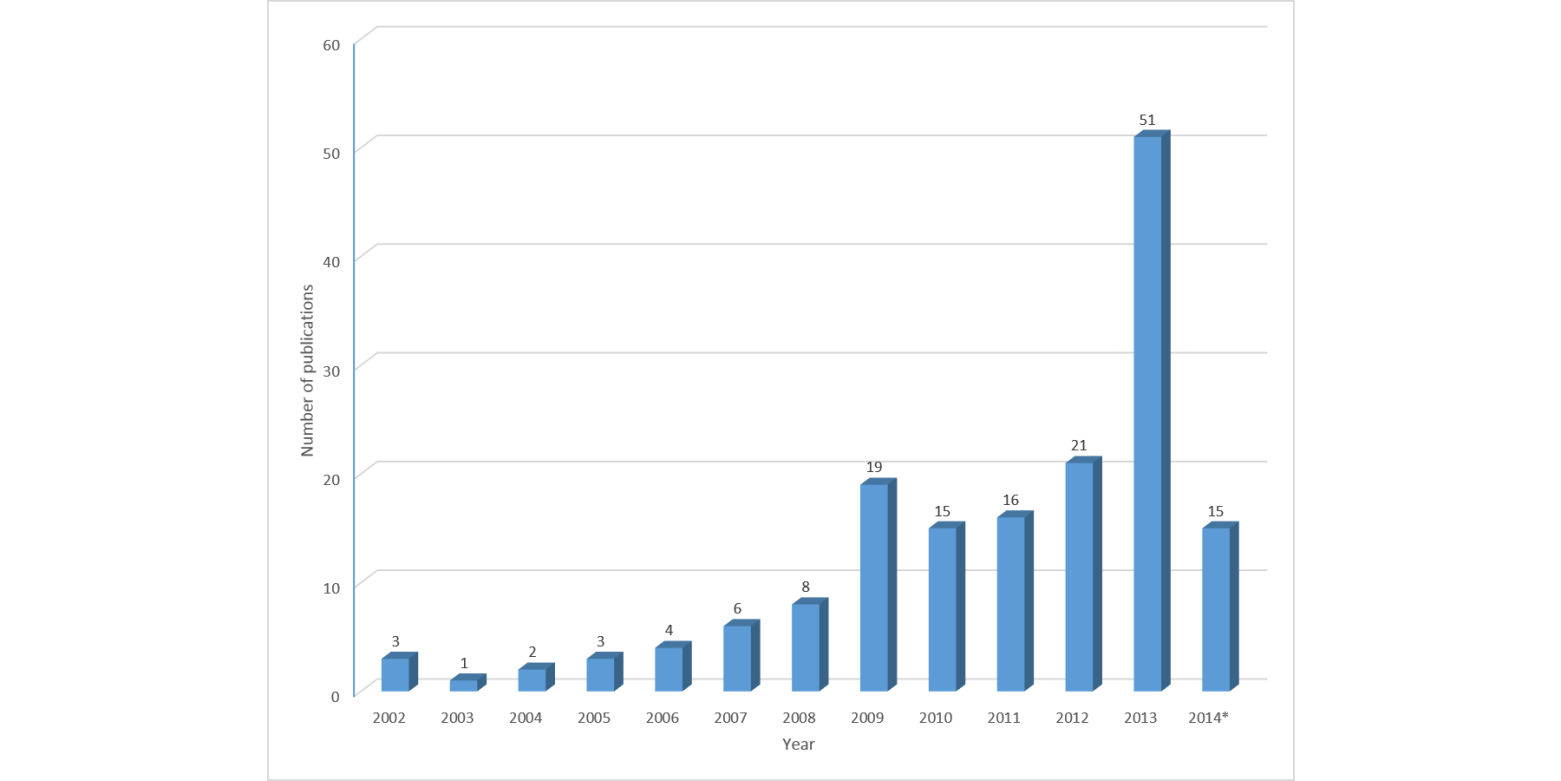
Number of publications per year on Internet interventions for depression in regular care settings. *The number for 2014 is up to March 24 only.

**Figure 3:**
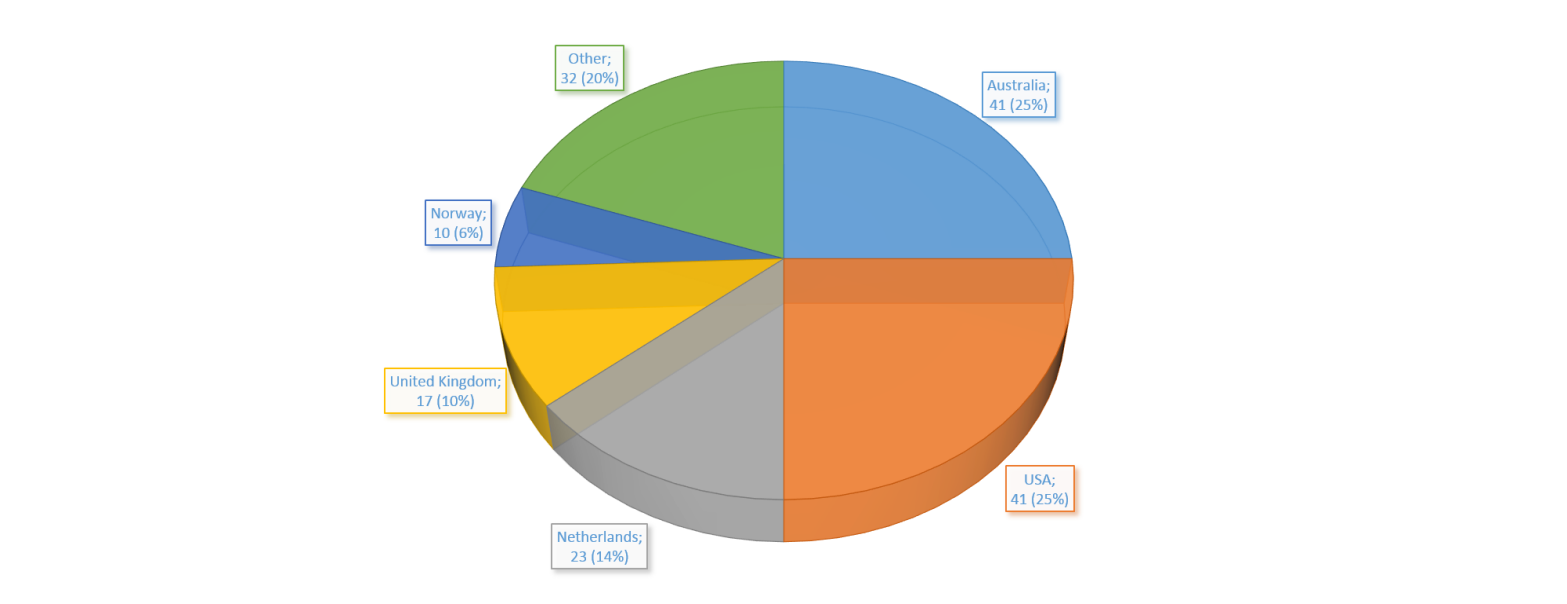
Number and percentage of publications on Internet interventions for depression in regular care settings, by country, 2002 to March 24, 2014.

Studies of Internet interventions for depression meeting our inclusion criteria were published from 2002 and up to the date of our final search on March 24, 2014. There was a modest increase in the number of publications during this period, with a marked increase and peak occurring in 2013 (*K*=51, 31.1%; Figure 2). Based on the first authors’ affiliation, the majority of publications originated from Australia and the United States (Figure 3). If categorized based on geographical region, Europe (*K*=69, 42.1%) generated most publications, followed by North America (*K*=52, 31.7%), Australia (*K*=41, 25.0%), and Asia (*K*=2, 1.2%).

### Implementation Components

Of the 164 included references, 122 (74.4%) were coded onto one or several of the implementation components, although none explicitly reported using the AIF. A Cochran Q test indicated a significant difference in the reporting of implementation components (*χ*^2^_7,N__=164_= 484.56, *P*<.001; see Table 1). Of the 122 references, 120 (98.4%) were coded on the competency drivers (ie, selection, training, coaching, and performance) and 13 (10.7%) on the organizational drivers (ie, administrative support, system intervention, and decision support); none of the references reported any information on aspects of leadership (Table 1). These results were also reflected in the total of 302 analysis units that were extracted, of which 281 (93.0%) units concerned the competency drivers and 21 (7.0%) units concerned the organizational drivers (Table 1).

**Table 1:** Number and percentage of references and units coded on the initial implementation components.

Implementation components	*K* ^a^	%	*k* ^b^	%
Selection	114	69.5	164	54.3
Training	28	17.1	44	14.6
Supervision	36	22.0	61	20.2
Performance	9	5.5	12	4.0
Decision support	2	1.2	4	1.3
Administrative support	7	4.3	8	2.6
System intervention	8	4.9	9	3.0
Leadership	0	0.0	0	0.0

^a^Unique references coded onto the various implementation components.

^b^Analysis units extracted from the references.

As Table 2 shows, of the 164 included references, most contained information pertaining to the selection of users for an intervention, few reported information relevant for practitioners, and almost none reported on higher levels. However, it is interesting to note that almost all information was related to the competency drivers (ie, selection, training, supervision, and performance assessments), while there was barely any information reported on the organizational drivers (ie, decision support, administration, system intervention, and leadership). This may explain the lack of information about the implementation at higher or across organizational levels (ie, beyond the practitioner level).

**Table 2:** Number and percentage of references (*K*) coded onto the initial implementation components across organizational levels.

Level	Selection, *K* (%)	Training, *K* (%)	Supervision, *K* (%)	Performance, *K* (%)	Decision support, *K* (%)	Administrative support, *K* (%)	System intervention, *K* (%)	Leadership, *K* (%)
User	100 (61.0)	8 (4.9)	28 (17.1)	2 (1.2)	0 (0.0)	1 (0.6)	1 (0.6)	0 (0.0)
Practitioner	13 (7.9)	14 (8.5)	15 (9.1)	8 (4.9)	1 (0.6)	1 (0.6)	1 (0.6)	0 (0.0)
Supervisor	0 (0.0)	0 (0.0)	0 (0.0)	0 (0.0)	0 (0.0)	0 (0.0)	0 (0.0)	0 (0.0)
Group	0 (0.0)	0 (0.0)	0 (0.0)	0 (0.0)	0 (0.0)	1 (0.6)	0 (0.0)	0 (0.0)
Leadership	0 (0.0)	0 (0.0)	0 (0.0)	0 (0.0)	0 (0.0)	0 (0.0)	0 (0.0)	0 (0.0)
Organization	1 (0.6)	0 (0.0)	0 (0.0)	0 (0.0)	2 (1.2)	4 (2.4)	3 (1.8)	0 (0.0)
Residual^a^	1 (0.6)	1 (0.6)	0 (0.0)	0 (0.0)	0 (0.0)	1 (0.4)	4 (2.4)	0 (0.0)

^a^References not accounted for by any of the organizational levels.

Similar to the results in Table 2, the extracted analysis units show that most of the reported information pertained to the user level (Table 3). In addition, it became clear that 283 (93.7%) of the analysis units were coded either on the user or the practitioner level (ie, the individual level). This shows that the included references did not take into account a multilevel perspective on Internet interventions or examined Internet interventions from an organizational perspective. Furthermore, 281 (93.0%) of the analysis units were also coded on one of the competency drivers. This suggests that key aspects of the overall performance of the organization itself, to support and assure the continuing implementation of an intervention, and the work of practitioners and supervisors has not been adequately addressed in the literature.

**Table 3:** Number and percentage of analysis units (*k*) coded onto the initial implementation components across organizational levels.

Level	Selection, *k* (%)	Training, *k* (%)	Supervision, *k* (%)	Performance, *k* (%)	Decision support, *k* (%)	Administrative support, *k* (%)	System intervention, *k* (%)	Leadership, *k* (%)	Total, *k* (%)
User	122 (40.4)	8 (2.6)	33 (10.9)	4 (1.3)	0 (0.0)	1 (0.3)	1 (0.3)	0 (0.0)	168 (55.6)
Practitioner	40 (13.2)	36 (11.9)	28 (9.3)	8 (2.6)	2 (0.7)	1 (0.3)	0 (0.0)	0 (0.0)	115 (38.1)
Supervisor	0 (0.0)	0 (0.0)	0 (0.0)	0 (0.0)	0 (0.0)	0 (0.0)	0 (0.0)	0 (0.0)	0 (0.0)
Group	0 (0.0)	0 (0.0)	0 (0.0)	0 (0.0)	0 (0.0)	1 (0.3)	0 (0.0)	0 (0.0)	1 (0.3)
Leadership	0 (0.0)	0 (0.0)	0 (0.0)	0 (0.0)	0 (0.0)	0 (0.0)	0 (0.0)	0 (0.0)	0 (0.0)
Organization	1 (0.3)	0 (0.0)	0 (0.0)	0 (0.0)	2 (0.7)	4 (1.3)	4 (1.3)	0 (0.0)	11 (3.6)
Residual^a^	2 (0.7)	0 (0.0)	0 (0.0)	0 (0.0)	0 (0.0)	1 (0.3)	4 (1.3)	0 (0.0)	7 (2.3)
Total	164 (54.3)	44 (14.6)	61 (20.2)	12 (4.0)	4 (1.3)	8 (2.6)	9 (3.0)	0 (0.0)	302

^a^Analysis units not accounted for by any of the organizational levels.

### Qualitative Synthesis

The implementation components served as a priori themes and were applied throughout the analysis to examine the characteristics of the implementation of Internet interventions for depression. After coding and revising the a priori themes, we broke the initial template down into 5 levels: (1) user, (2) practitioner, (3) group, (4) organization, and (5) residual. However, it was clear that many themes counted fewer than 10 references, indicating narrow thematic structures. Thus, we reduced the development of the final template to 2 levels and produced 5 main themes: (1) guided support and (2) user recruitment, both subsumed under the user level, and practitioner (3) qualifications, (4) training, and (5) supervision (Table 4) [16,40-52]. It is important to note that, despite the emergence of these themes, the units that these themes comprise were typically global and scarcely described in the original text. For example, Clarke et al [53] stated that they “employed the HMO’s [health maintenance organization’s] electronic medical record,” with no further information on how they were given access to the health maintenance organization’s medical records (ie, important system knowledge on access to the target population).

**Table 4:** The final template with meaningful themes, corresponding codes, definitions, and examples.

Level	Theme	1st-level code	2nd-level code	*K* ^a^	%	Definition	Example
**1. User**				110	67.07			
	1.1. Guided support			26	15.85	An Internet-based self-help program including minimal, but regular, human involvement and support.	“Program coaches...provided motivational support to participants and clarified information contained within the program” [40].
		1.1.1. Program usage		15	9.15	Human support with guidance and direction on how to work through the intervention and its activities.	“…a weekly 10-minute telephone call from a telephone counselor. The purpose of these calls was to address any issues associated with the participants’ use of the intervention” [41].
	1.2. Recruitment			101	61.59	Activities related to promoting and advertising the intervention to potential end users.	“Individuals were spontaneous visitors from around the world to an automated internet-delivered program (e-couch)” pg 344 in [42]
		1.2.1. Direct-to-consumer marketing		88	53.66	Efforts to promote the intervention directly to end users.	“Callers to Lifeline’s 24 hour telephone counselling service in four major Australian cities were invited to participate in the trial by a telephone counsellor either during or at the conclusion of a counselling call” pg 2 in [43]
			1.2.1.1. Multichannel marketing	23	14.02	Efforts to promote the intervention directly via multiple platforms or communication channels.	“…recruited through press releases, banners and advertisements on the Internet, advertisements in magazines, referral by school doctors, through brochures and posters in schools, and through information to parents who are treated in mental health care” pg 2 in [44].
			1.2.1.2. Online	28	17.07	Efforts to promote the intervention directly using digital technologies mainly via the Internet.	“Paid advertising with Google was directed at people who had searched for a short questionnaire online to find out whether they had depression” pg 412 in [45].
			1.2.1.3. Print media	21	12.80	Efforts to promote the intervention directly by means of physical publications.	“…recruited...via a screening survey posted to 70,000 adults randomly selected from the electoral rolls of eight Australian electoral divisions (4 rural, 4 metropolitan)” pg 61 in [46].
		1.2.2. Professional referral		22	13.41	Transfer or direction of end users for an intervention, both directly and indirectly, by health professionals or treatment providers.	“Participants were recruited from 11 General Practices...A member of each of the eleven participating GP [general practitioner] Practices identified searched their patient record system to identify patients…Following identification of appropriate patients, a study information pack was sent by the practice” pg 642 in [47]
			1.2.2.1. In-person	15	9.15	Direct transfer of end users for an intervention by health professionals or treatment providers.	“Two general practitioners and two psychologists, all in Sydney (Australia), referred individuals with symptoms of depression to the first author” pg 2 in [48].
**2. Practitioner**				46	28.05		
	2.1. Qualifications			37	22.56	Formal and informal background education or training, or both, among practitioners delivering Internet interventions.	“Psychological support can be provided by the following: • Graduate mental health worker • Practice nurse • GP • Assistant psychologist • Care worker • Other mental health professionals (Administrators/receptionists can offer some support to ensure that users are set up correctly on the programme but not psychological support)” pg 21 in [16].
		2.1.1. Therapists		20	12.20	A practitioner formally qualified and trained in psychological treatment methods.	“Coaches differed in their level of formal training, ranging from master’s level psychology students (n=1) and psychotherapists-in-training (n=1) to experienced CBT [cognitive behavioral therapy]-trained psychotherapists with more than 10 years of professional experience (n=3)” [49].
	2.2. Training			21	12.80	Acquisition of new knowledge, skills, and abilities required to work with Internet interventions.	“Therapists were given training and a treatment manual containing a broad guide of how to respond in their reviews (supporting progress, giving encouragement, specific feedback on activities shared)” [50].
		2.2.1. Method		11	6.71	Prescribed practice or process of acquiring new knowledge, skills, and abilities needed to produce desired outcomes with the Internet intervention.	“Training involved a mix of didactics and roleplay around conducting functional analysis in perinatal-specific domains with the chief investigator (H.O.), a clinical psychologist with specialty expertise in BA [behavioral activation] and perinatal depression, and an IAPT [Improving Access to Psychological Therapies] trainer (J.W.)” pg 3 in [51].
	2.3. Supervision			11	6.71	Coaching of practitioners working with users through some form of on-the-job training.	“Comments made by the health care staff to participants in the e-mail sessions were discussed beforehand … with the CBT specialists.” pg 497 in [52].

^a^Number of references coded on a theme or subtheme.

#### Guided Support (1.1)

Guided support emerged as 1 of 2 main themes among users derived from the a priori coaching theme, where users were provided support online or by telephone, most often by a therapist. These contact points were typically regular (eg, weekly), therapist initiated, and brief (eg, 10–20 minutes). Guided support usually involved varying forms of nonclinical supervision, such as technical support (*K*=1), preparations for general practitioner visits (*K*=1), and help with homework assignments (*K*=1). Most often, however, it was not possible to determine the exact purpose, methods, or contents of guided support.

##### Program Usage (1.1.1)

Program usage emerged as the only subtheme under guided support. This specific type of support was related to direction and guidance of users on how to work through the intervention and its activities. In other words, program usage was concerned with assuring fidelity to the intervention. There were two ways of providing support for program usage: either by (1) attending an introductory course or being briefed by practitioners early on about the operations of the intervention, or (2) receiving ongoing help when facing any problems with the intervention or certain tasks.

#### Recruitment (1.2)

Recruitment was derived from the a priori selection theme that was concerned with activities related to selecting end users, practitioners, and organizations to use or work with an intervention. However, Table 3 shows, 122 (74.4%) of 164 units were related to end users. Thus, information initially pertaining to selection across multiple organizational levels was conceptualized as user recruitment and divided in 2 first-level subthemes: (1) direct-to-consumer marketing (DTC) and (2) professional referral.

##### Direct-to-Consumer Marketing (1.2.1)

DTC marketing refers to recruitment activities aimed directly at end users (ie, the consumer). There was a wide range of DTC recruitment strategies, from counseling services (*K*=4) to organizations (*K*=2) and school settings (*K*=2). However, a multichannel strategy, online recruitment, and recruitment using print media were most common and emerged as second-level subthemes.

###### Multichannel Marketing (1.2.1.1)

Multichannel DTC marketing involved the combination of two or more recruitment strategies. The studies using multichannel marketing often recruited users from a wider population and used more targeted marketing efforts. For example, in a study by Haga and colleagues [54], the researchers recruited pregnant women through midwives and public health nurses in well-baby clinics and hospitals, who, in turn, handed out brochures about the study and intervention. At the same time, pregnant women were also recruited through social media (ie, Facebook).

###### Online (1.2.1.2)

Online DTC recruitment strategies have mainly consisted of using and testing ads and banners, such as in the study of Barrera et al [55]. They examined the impact of Spanish and English keywords for a Google AdWords campaign to recruit pregnant women and found that broad descriptive words related to pregnancy, health, and distress resulted in higher international enrollment rates. For most recruitment strategies, however, more geographically targeted online advertisements may be necessary, as investigated by Jones and colleagues [56]. Interestingly, they found that between one-third and half of the ads were wrongly targeted by AdWords to nearby postal code areas. In a follow-up study [57], AdWords location targeting was still found to be more effective than posting ads at local organization websites, despite the misdirected ads. Organization websites may still be effective but need to be advertised through trustworthy, relevant, and familiar mental health organizations [58].

###### Print Media (1.2.1.3)

Use of print media consisted of ads and articles in national and local newspapers, and invitation letters by postal mail (eg, questionnaires, brochures, or study information). In contrast to multichannel marketing, marketing through print media mostly recruited users from the general public. Only 3 studies using print media appear to have used a more targeted approach [47,59,60]. For example, in the study of Woodford et al [47], general practices searched their patient records to identify patients with a diagnosis of depression or who may have experienced mild to moderate depression over the last 6 months.

##### Professional Referral (1.2.2)

Professional referral is the second first-level subtheme that emerged under the main theme selection. Referrals were either a part of a multichannel marketing strategy or, most often, direct in-person referrals to an intervention. According to the AIF, routines for referral are usually related to system intervention because they entail collaboration with external agencies such as general practitioners. However, none of the articles using referrals contained any information on how interorganizational agreements and routines for referrals were established or evaluated, or how these were embedded in the larger system. Thus, we coded and analyzed these units only as a form of recruitment procedure.

###### In-Person (1.2.2.1)

Direct in-person referrals were most common where, for instance, patients were prescribed an Internet intervention directly by their general physician or mental health specialist (eg, see [61]).

#### Qualifications (2.1)

We identified qualifications as the first main theme among practitioners, derived from the selection theme. Of the 38 studies, 36 (94.7%) involved practitioners that either had a completed college or university degree or were in their last year of a formal training program, most of whom were therapists (see below). In 11 (28.9%) of the 38 studies, interventions were delivered by medical staff consisting of either nurses or general practitioners, or both, and, in 7 (18.4%) studies, interventions were delivered by various practitioners such as school teachers, mental health workers, or occupational health staff. Interestingly, according to 2 (5.3%) studies, such formal qualifications may not be necessary, and it appears that laypersons may administer Internet interventions just as effectively as therapists or mental health workers [62,63].

##### Therapists (2.1.1)

Of the 38 studies, 20 (52.6%) reported the use of therapists to deliver the interventions. Psychologists participated in 13 (34.2%) studies and an additional 2 (5.3%) studies involved psychologists in combination with other mental health professionals. The remaining 5 (13.5%) studies either involved mental health workers or did not specify the therapists’ formal training background.

#### Training (2.2)

Training emerged as the second main theme among practitioners, with 1 subtheme relating to how practitioners were trained in the administration of the Internet intervention (ie, method; Table 4). Of the remaining studies, 9 (23.7%) of 21 reported on the scope of training (ie, ranging from brief 1-hour training sessions to 5 days of training); 4 (17.1%) reported providing special training in the skills required to administer the Internet intervention, such as electronic, text-based communication (eg, see pg 210 in [64]); 3 (14.3%) mentioned that training was provided by either the intervention developers or the principal study investigator; and 1 (4.8%) arranged educational sessions. In addition, 4 (17.1%) studies noted in passing that practitioners received training, but without providing any further information.

##### Method (2.2.1)

A variety of methods were used to train practitioners. Of 11 studies, 4 (36.4%) reported the use of video demonstrations (eg, see pg 186 in [65]). In 3 (27.2%) other studies, practitioners reviewed the contents of the intervention in order to adequately address participants’ questions and provide assistance with tasks and activities. Of 11 studies, 2 (18.1%) used a mix of didactics and practice in, for example, cognitive behavioral skills or responding to clinical emergencies (eg, pg 743 in [63]). Also, 1 (9.1%) study had a specialist in cognitive behavioral therapy provide a 3-hour lecture about it to practitioners, while 1 (9.1%) study provided newly educated psychologists with additional training in delivering the specific treatment manuals.

#### Supervision (2.3)

Supervision was the third main theme among practitioners that we derived from the initial coaching component. Information was mostly concerned with *the who* (*K*=8, 44.5%) and *the extent* (*K*=6, 33.3%) of supervision. That is, supervision was mostly provided regularly (eg, weekly) by psychologists or therapists. Beyond that, supervision was used for case management in 3 (16.7%) of the 18 studies (eg, discuss practitioners’ response to users in email sessions; see pg 497 in [52]), and 1 study reported using supervision to develop interview scripts for users [66].

## Discussion

### Principal Findings

The aim of this study was twofold: first, to examine whether there are any systematic differences in the implementation of Internet interventions for depression in the literature in terms of core implementation components and, thereby, identify any knowledge gaps; and second, to examine what characterizes the implementation in the literature. In total, we identified 164 references, of which 122 (74.4%) were coded onto the AIF. Overall, the results show that no studies had any hard data about which components are critical for implementation and which components may be adapted without compromising intervention outcomes in regular practice. Information related to the competency drivers (ie, selection, training, and supervision) was most frequently reported; however, in terms of the organizational drivers, fewer than 10 references were coded onto decision support, administrative support, and system intervention. No studies contained any information related to leadership.

### Competency Drivers

Our results revealed that studies concerned with selection were focused on the recruitment of users for the intervention or study, rather than on finding the right personnel or organizations to carry out or support the new intervention [34]. This likely reflects a common practice of reporting on participant recruitment in studies (eg, see the CONSORT statement [67]), rather than that research has been genuinely concerned with investigating various recruitment strategies. This is supported by the few studies identified to actually investigate recruitment processes (eg, see [55,56]). Nevertheless, print media and online recruitment strategies were typically used, although a multichannel marketing strategy was most common. The predominance of DTC strategies (ie, self-referrals) supports the notion that Internet interventions for depression have yet to become an integral part of routine care.

Our review found that staff selection has not been studied, but that almost all practitioners had higher education in psychological, medical, or other health sciences. We also found that formal qualifications may not be necessary to administer Internet interventions effectively. However, regardless of qualifications, a strong and active implementation strategy, which integrates and addresses all of the implementation components, is important to maintain their quality and effectiveness [68]. As such, a lack of formal qualifications and practitioner heterogeneity may be compensated for by, for example, receiving high-quality training and supervision from highly competent and experienced practitioners. Training was, however, typically brief and consisted of lectures, videos, and written materials (eg, program review or treatment manuals). This does not tell much about the quality of training, and it can be argued that the technology does most of the work of delivering the interventions and, thus, extensive and complex training may not be required. However, a few small-scale studies have shown that brief training is insufficient to sustain changes in practice over time [14,15], while previous studies have demonstrated that frequently used training methods, such as reviewing treatment manuals, are not necessarily efficient for acquiring a new set of skills [69,70].

Supervision may compensate for brief training and has been shown to increase practitioner behavior change [71]. This may be particularly true when regular, ongoing supervision is provided by highly educated and experienced supervisors, which makes it possible for practitioners to embed new clinical skills into their existing repertoire and ongoing work. This may, however, depend on highly qualified and skilled supervisors and the methods that are used during supervision. There are many various labels for supervision, such as consultation [71], coaching [72], and auditing [73]. However, such implementation strategies were rarely defined, and often inadequately described in the literature. This also seemed to apply to guided support, which emerged as a theme among end users. Thus, it remains unclear what therapist support consisted of, and it appears that the contents of the therapist support that is provided are heterogeneous. Of 31 (18.9%) studies, only 1 subtheme—program usage—emerged. We could not determine whether this is because the purpose and clinical guidelines for therapist support are inadequately described in the literature, or whether the heterogeneity in therapist support is real.

### Organizational Drivers

It is important to acknowledge that competency drivers do not exist in a vacuum, but rely on and are supported by an organization that provides management and administrative structures, and relates to external systems (ie, service delivery models), all of which can affect the implementation. However, there were no emerging themes among any of the organizational drivers, despite the importance of components, such as decision-support systems and leadership, for improving clinical practice [74,75]. This does not mean that systems for decision making do not exist or that aspects of leadership have not been addressed in practice. It simply reflects that no studies have properly assessed these implementation components or implementation processes and quality more generally. Some studies did, however, report information related to administrative support (*K*=7) and system intervention (*K*=8), although there was insufficient information for any themes to emerge. However, Andersson and Hedman [19] identified several issues related to the implementation of iCBT in practice, and which are highly relevant for the administration of Internet interventions and system-related work with external agencies: (1) data security, (2) robust Web solutions, (3) online assessment procedures (including diagnostic interviews), (4) referral routes, patient management, and outcome monitoring, (5) the role of professional organizations, and (6) development of clearly formulated policies, procedures, and practice guidelines (see also [14]). In addition, Titov and colleagues [22] suggested technical support and legislation across federal, state, and international laws as barriers that become more actualized with Internet interventions. Most of these issues, however, have not been studied, except for referrals, in particular self-referrals, which are likely to affect the uptake of and, possibly, adherence to iCBT [76,77].

### General Discussion

It is important to establish a robust evidence base for Internet interventions, but it is equally important to establish a robust evidence base for the delivery of Internet interventions in practice, by moving beyond studies of efficacy and effectiveness to implementation. This is a necessary step to scale up the dissemination and integration of Internet interventions in routine practice, and ultimately to provide better and safer health care services. Limited reporting on the different implementation components limits the value of these studies for decision makers and other stakeholders, as most of the studies did not include sufficiently relevant information to understand how to translate these results into practice. The lack of emphasis on organizational drivers, in particular, may impede effective implementation. Thus, stakeholders have to rely on and become dependent on the know-how of the relatively few communities working with Internet-based prevention and treatment of depression (ie, Australia, United States, the Netherlands, Sweden, United Kingdom, Canada, and Norway).

Implementation of Internet interventions in routine care is still in its infancy, and there is no strong evidence or methods for transferring Internet-based prevention and treatment to service delivery settings (see also [78]). Service delivery models have not been adequately developed or tested, even though governments and professional societies in several countries are recommending Internet interventions in their national guidelines (eg, Australia [79], United Kingdom [80], Sweden [81], and Norway [82]). Yet the obvious question is how to integrate Internet interventions into new or existing health care services for large-scale implementation [22]. Stepped-care models and other service delivery models have been proposed [23,25] but should be appraised critically. Technological advances and novel practices may not fit with existing models of health care service delivery and may need to be redefined [21]. Already in 2009, Bennett and Glasgow noted [20] that there has been relatively little discussion of contextual issues in eHealth. The results of our review suggest that this has not changed much since then. To date, studies have largely focused on testing the effects of Internet interventions on users, while more work remains to understand the organizational, systemwide, and contextual features of implementation. Thus, the important future lessons for Internet interventions are really those concerning the knowledge transfer from science to practice. This will support governments, researchers, and other stakeholders in implementing effective Internet interventions in practice, replicating studies, conducting independent research, building competence, and driving development of Internet interventions. Currently, however, Governments, researchers, and others are dependent on the few existing experts and research milieus in this field for the implementation of Internet interventions.

### Strengths and Limitations

This study has several strengths and limitations. First, we used the scoping review methodology, which is effective in mapping the state-of-the-art and identifying gaps in the literature. However, in line with the methodology, we emphasized the breadth rather than depth of knowledge and did not assess the quality of the included studies [83].

Second, we constructed a comprehensive search strategy, but Internet interventions and implementation are relatively new areas of scientific inquiry. Thus, there is a tremendously wide range of terminology, which prevented us from combining search terms for Internet interventions and depression with implementation, which produced a large and unmanageable number of search results. Furthermore, the terms efficacy and effectiveness are sometimes used interchangeably, and it may be difficult to distinguish whether unguided interventions should be classified as efficacy or effectiveness trials. In contrast to guided interventions, unguided interventions may be offered directly to users from university clinics, private companies, or online, without being implemented in a health care setting (eg, see [84]). Consequently, we may have missed some relevant articles or included some efficacy trials.

Third, the extensive number of included studies and text material in this scoping review also means that we, most likely, have missed information relevant for implementation. This is mainly because none of the studies were de facto implementation studies, which means that much of the reported information has been scarce and thereby ambiguous. This has occasionally made it difficult to identify, assess, and code information from the studies, and has, most certainly, left some information unidentified or incorrectly classified and analyzed. However, the extensive number of studies and text material also means that a larger number of studies and data would be needed for substantial changes to occur in the results.

Fourth, we did not consider that publications are nested within authors. Different authors may emphasize and report on different aspects of the implementation of Internet interventions, and publish several articles based on 1 study. An author may also vary in his or her influence on publications depending on their role and contribution (eg, principal investigator versus supervisor).

Fifth, we used Fixsen and colleagues’ [34] AIF. Other implementation theories and models may have identified other types of relevant information and thus provided different results. According to Tabak et al [85], the AIF is more concerned with integrating evidence-based practices within a setting than with disseminating them to the target audience via determined channels using a planned strategy. One of the strengths of this study, however, is that we applied the AIF in a flexible manner and revised the model through the template analysis. As our results show, dissemination of evidence-based practices to target audiences is not adequately addressed in the AIF, which resulted in recruitment emerging as a main theme at the user level. Furthermore, the AIF does not operate at the policy level or higher socioecological levels (eg, government). The conclusions in this review must be interpreted in the light of these limitations.

### Future Research Directions

This scoping review has highlighted several important issues for future research. First, for Internet interventions for depression to become more widespread and embedded in regular practice, it is necessary to move from studies of efficacy and effectiveness to implementation. There is a clear need for more primary implementation studies that are based on clearly defined models and theories of implementation (for overview, see [85]), and preferably that link implementation outcomes with intervention outcomes. This will help distinguish between what is known about an effective treatment (ie, studies of effectiveness) and what is actually benefiting clients and providing safer and better health care services (ie, implementation [86]).

Second, there is an urgent need to improve reporting guidelines such as the eHealth CONSORT statement [87]. The lessons learned about implementation in randomized trials and other studies can increase sharply, simply by requiring that future studies regularly and systematically describe the implementation of the intervention.

Third, there is a need for experimental studies on the effects of specific implementation strategies—for example, which formal and informal qualifications are important for administration of Internet interventions among practitioners or what recruitment strategies are likely to be more efficient. However, probably the most striking gap in the literature is the lack of investigation of the interaction *between* the different implementation components and their relative influence *over time* [34]—for example, the relationship between the amount of training and duration of supervision necessary to administer an intervention in a competent and skillful manner.

Fourth, more research is needed at different organizational levels of implementation, including leadership and management practices. Much of the available information on implementation pertains to end users, and much less is known about the practitioners, organizations, and systems within which interventions are embedded.

Fifth, we would encourage authors and journals to routinely publish implementation protocols similar to study or intervention protocols. This would provide a greater understanding of *what* activities are necessary and *how* these activities need to be carried out to (re-)produce the achieved results from any given trial. It is, however, also important that implementation protocols use standardized reporting guidelines such as the assessment of transferability and adaptation of health promotion interventions (ASTAIRE) [88] to, among other things, ensure that they are directly applicable in practice. Implementation protocols may help explain why some intervention trials succeed and others do not and, most important, they would support independent research and knowledge transfer between different research communities and contexts.

### Conclusions

This review aimed at investigating what is known about the implementation of Internet interventions for depression in the literature. Overall, the results showed that limited emphasis has been given to their implementation in practice and that leadership and organizational drivers have been largely neglected. Recruiting users for the interventions was, by far, most commonly reported and typically carried out by the use of print media, online recruitment, or multichannel marketing strategies and, to some extent, professional referrals. Therapist support to ensure program usage was also characteristic of Internet interventions and, although brief training and regular supervision may be sufficient for administering Internet interventions, more research is needed.

The Internet holds promise as an effective platform for the delivery of interventions for depression. However, to progress and make Internet interventions more widely available, it is of utmost importance that the field prioritize implementation practice and research, and move beyond studies of efficacy and effectiveness. Only by allocating research efforts to implementation may the field be able to provide stakeholders and decision makers with knowledge of which strategies promote effective implementation and, consequently, provide better health services for the target population.

## Appendix

Multimedia Appendix 1 Search strategy.

Multimedia Appendix 2 Complete list of hand-searched journals.

Multimedia Appendix 3 List of references included for analysis.

## Abbreviations

AIF: active implementation framework
ASTAIRE: assessment of transferability and adaptation of health promotion interventions
DTC: direct-to-consumer marketing
iCBT: Internet-based cognitive behavior therapy
IGLO: individual, group, leadership, and organizational levels
