# Supplementary material for: The Implementation of Internet Interventions for Depression: A Scoping Review
Source: J Med Internet Res. 2016 Sep 8;18(9):e236. doi: 10.2196/jmir.5670 (PMC5034149; doi:10.2196/jmir.5670)
Supplement: Multimedia Appendix 1 [file jmir_v18i9e236_app1.pdf]

MULTIMEDIA APPENDIX 1: SEARCH STRATEGY

Ovid MEDLINE(R) In-Process & Other Non-Indexed Citations and Ovid MEDLINE(R) 1946 to Present (March 24, 2014)

#	Searches	Results
1	exp Internet/	48404
2	(ehealth* or e-health* or emental health* or e-mental health* or e-therap* or e-psycholog* or web or website* or internet* or online* or consumer health application* or cybertherap* or cyberpsycholog*).tw.	106328
3	1 or 2	124297
4	exp Therapeutics/	3299774
5	exp Psychotherapy/	147509
6	(intervention* or treat* or therap* or psychotherap* or program* or self-care or selfcare or self-management or self-help or selfhelp or self-control or counsel* or education* or training or supervision).tw.	5589214
7	4 or 5 or 6	7393847
8	exp Depression/ or exp Depressive Disorder/	148943
9	depress*.tw.	310073
10	8 or 9	346505
11	3 and 7 and 10	1935
