# Supplementary material for: The Implementation of Internet Interventions for Depression: A Scoping Review
Source: J Med Internet Res. 2016 Sep 8;18(9):e236. doi: 10.2196/jmir.5670 (PMC5034149; doi:10.2196/jmir.5670)
Supplement: Multimedia Appendix 2 [file jmir_v18i9e236_app2.pdf]

MULTIMEDIA APPENDIX 2: COMPLETE LIST OF HAND-SEARCHED JOURNALS

No.	Journal
1	Addiction
2	Administration and Policy in Mental Health
3	American Journal of Community Psychology
4	American Journal of Preventive Medicine
5	Annals of Behavioral Medicine
6	Australian and New Zealand Journal of Psychiatry
7	Behaviour Research and Therapy
8	Behavioural and Cognitive Psychotherapy
9	BioMed Central
10	BMC Psychiatry
11	British Journal of Psychiatry
12	Cognitive Behaviour Therapy
13	Computers in Human Behavior
14	Dissertation Abstracts International: Section B
15	European Psychiatry
16	Health Education Research
17	Implementation Science
18	Interactive Journal of Medical Internet Research
19	Iproceedings
20	JMIR Cancer
21	JMIR Challenges
22	JMIR Diabetes
23	JMIR Human Factors
24	JMIR Medical Education
25	JMIR Medical Informatics
26	JMIR Mental Health
27	JMIR mHealth and uHealth
28	JMIR Preprints
29	JMIR Public Health and Surveillance
30	JMIR Rehabilitation and Assistive Technologies
31	JMIR Research Protocols
32	JMIR Serious Games
33	Journal of Affective Disorders
34	Journal of Applied Psychology
35	Journal of Clinical Psychology
36	Journal of Consulting and Clinical Psychology
37	Journal of Medical Internet Research
38	Medical Journal of Australia
39	Medicine 2.0
40	PLoS ONE
41	Preventive Medicine
42	Psychiatrische Praxis
43	Psychological Medicine
44	The Journal of Positive Psychology
45	The Journal of Primary Prevention
46	The Sciences and Engineering
