# Supplementary material for: The Implementation of Internet Interventions for Depression: A Scoping Review
Source: J Med Internet Res. 2016 Sep 8;18(9):e236. doi: 10.2196/jmir.5670 (PMC5034149; doi:10.2196/jmir.5670)
Supplement: Multimedia Appendix 3 [file jmir_v18i9e236_app3.pdf]

MULTIMEDIA APPENDIX 3: LIST OF INCLUDED REFERENCES FOR ANALYSIS

ID	First author	Publication year	Title	Journal
1	Allen, M	2008	Improving patient-clinician communication about chronic conditions - Description of an Internet-based nurse e-coach intervention	Nursing Research
2	Almlov, J	2009	Therapist factors in Internet-delivered cognitive behavioural therapy for major depressive disorder	Cognitive Behaviour Therapy
3	Bae, J	2009	Development of a user-centered health information service system for depressive symptom management	Nursing & Health Sciences
4	Barazzone, N	2012	Computerized cognitive behavioural therapy and the therapeutic alliance: A qualitative enquiry	British Journal of Clinical Psychology
5	Barrera, A Z	2014	Keywords to recruit Spanish- and English-speaking participants: evidence from an online postpartum depression randomized controlled trial	Journal of Medical Internet Research
6	Batterham, P J	2008	Predictors of adherence among community users of a cognitive behavior therapy website	Patient Preference & Adherence
7	Beattie, A	2009	Primary-care patients' expectations and experiences of online cognitive behavioural therapy for depression: a qualitative study	Health Expectations
8	Bendelin, N	2011	Experiences of guided Internet-based cognitive-behavioural treatment for depression: a qualitative study	BMC Psychiatry
9	Boggs, J. M.	2014	Web-based intervention in mindfulness meditation for reducing residual depressive symptoms and relapse prophylaxis: a qualitative study.	Journal of Medical Internet Research
10	Bolier, L	2013	An internet-based intervention to promote mental fitness for mildly depressed adults: randomized controlled trial	Journal of Medical Internet Research
11	Bowie, C R	2013	Cognitive remediation for treatment-resistant depression: effects on cognition and functioning and the role of online homework	Journal of Nervous and Mental Disease
12	Button, K S	2012	Factors associated with differential response to online cognitive behavioural therapy	Social Psychiatry and Psychiatric Epidemiology
13	Calear, A L	2009	The YouthMood Project: a cluster randomized controlled trial of an online cognitive behavioral program with adolescents	Journal of Consulting and Clinical Psychology
14	Calear, A L	2013	Adherence to the MoodGYM program: outcomes and predictors for an adolescent school-based population	Journal of Affective Disorders
15	Carper, M M	2013	The Dissemination of Computer-Based Psychological Treatment: A Preliminary Analysis of Patient and Clinician Perceptions	Administration and Policy in Mental Health and Mental Health Services Research
16	Christensen, H	2002	Web-based cognitive behavior therapy: analysis of site usage and changes in depression and anxiety scores	Journal of Medical Internet Research

17	Christensen, H	2004	A comparison of changes in anxiety and depression symptoms of spontaneous users and trial participants of a cognitive behavior therapy website	BMJ: British Medical Journal (International Edition)
18	Christensen, H	2004	Delivering interventions for depression by using the Internet: randomised controlled trial	Journal of Medical Internet Research
19	Christensen, H	2006	Free range users and one hit wonders: community users of an Internet-based cognitive behaviour therapy program	Australian and New Zealand Journal of Psychiatry
20	Christensen, H	2006	Online randomized controlled trial of brief and full cognitive behaviour therapy for depression	Psychological Medicine
21	Christensen, H	2007	Reaching standards for dissemination: a case study	Studies in Health Technology and Informatics
22	Clarke, G	2002	Overcoming depression on the Internet (ODIN): a randomized controlled trial of an Internet depression skills intervention program	Journal of Medical Internet Research
23	Clarke, G	2005	Overcoming Depression on the Internet (ODIN) (2): a randomized trial of a self-help depression skills program with reminders	Journal of Medical Internet Research
24	Clarke, G	2009	Randomized effectiveness trial of an Internet, pure self-help, cognitive behavioral intervention for depressive symptoms in young adults	Cognitive Behaviour Therapy
25	Cook, J E	2002	Working alliance in online therapy as compared to face-to-face therapy: Preliminary results	CyberPsychology & Behavior
26	Crisp, D	2014	An online intervention for reducing depressive symptoms: Secondary benefits for self-esteem, empowerment and quality of life	Psychiatry Research Feb
27	Currie, S L	2010	Development and usability of an online CBT program for symptoms of moderate depression, anxiety, and stress in post-secondary students	Computers in Human Behavior
28	Danaher, B G	2012	Web-Based Intervention for Postpartum Depression: Formative Research and Design of the MomMoodBooster Program	JMIR Research Protocols
29	Danaher, B G	2013	MomMoodBooster web-based intervention for postpartum depression: feasibility trial results	Journal of Medical Internet Research
30	De Graaf, L E	2009	Clinical effectiveness on online computerised cognitive-behavioural therapy without support for depression in primary care: Randomised trial	The British Journal of Psychiatry
31	de Graaf, L E	2009	Use and acceptability of unsupported online computerized cognitive behavioral therapy for depression and associations with clinical outcome	Journal of Affective Disorders
32	de Graaf, L	2010	Predicting outcome in computerized cognitive behavioral therapy for depression in primary care: A randomized trial	Journal of Consulting and Clinical Psychology
33	de Graaf, L E	2011	One-year follow-up results of unsupported online computerized cognitive behavioural therapy for depression in primary care: A randomized trial	Journal of Behavior Therapy and Experimental Psychiatry

34	Department of Health	2007	Improving Access to Psychological Therapies (IAPT) Programme: Computerized Cognitive Behavioural Therapy (cCBT) implementation guidance	Department of Health
35	Donker, T	2013	Internet-delivered interpersonal psychotherapy versus internet-delivered cognitive behavioral therapy for adults with depressive symptoms: randomized controlled noninferiority trial	Journal of Medical Internet Research
36	Donker, T	2013	Predictors and moderators of response to internet-delivered Interpersonal Psychotherapy and Cognitive Behavior Therapy for depression	Journal of Affective Disorders
37	Ebert, D D	2013	For Whom Does It Work? Moderators of Outcome on the Effect of a Transdiagnostic Internet-Based Maintenance Treatment After Inpatient Psychotherapy: Randomized Controlled Trial	Journal of Medical Internet Research
38	Eisen, J C	2013	Pilot study of implementation of an internet-based depression prevention intervention (CATCH-IT) for adolescents in 12 US primary care practices: Clinical and management/organizational behavioral perspectives	Primary Care Companion to the Journal of Clinical Psychiatry
39	Farrer, L	2011	Internet-based CBT for depression with and without telephone tracking in a national helpline: randomised controlled trial	PLoS ONE
40	Farrer, L	2012	Web-based cognitive behavior therapy for depression with and without telephone tracking in a national helpline: secondary outcomes from a randomized controlled trial	Journal of Medical Internet Research
41	Farrer, L M	2013	Predictors of adherence and outcome in internet-based cognitive behavior therapy delivered in a telephone counseling setting	Cognitive Therapy and Research
42	Gerhards, S A	2011	Improving adherence and effectiveness of computerised cognitive behavioural therapy without support for depression: a qualitative study on patient experiences	Journal of Affective Disorders
43	Gerhards, S A H	2011	Economic evaluation of online computerized cognitive behavioural therapy without support for depression in primary care: A randomized trial	Journal of Mental Health Policy and Economics
44	Gerrits, R S	2007	Master your mood online: A preventive chat group intervention for adolescents	Australian e-Journal for the Advancement of Mental Health
45	Gladstone, T	2014	Understanding adolescent response to a technology-based depression prevention program	Journal of Clinical Child and Adolescent Psychology
46	Gun, S Y	2011	Acceptability of Internet treatment of anxiety and depression	Australasian Psychiatry
47	Hadjistavropoulos, H D	2012	Dissemination of therapist-assisted internet cognitive behaviour therapy: development and open pilot study of a workshop	Cognitive Behaviour Therapy

48	Haga, S M	2013	Mamma mia: a feasibility study of a web-based intervention to reduce the risk of postpartum depression and enhance subjective well-being	JMIR Research Protocols
49	Hedman, E	2014	Effectiveness of Internet-based cognitive behaviour therapy for depression in routine psychiatric care	Journal of Affective Disorders
50	Hickie, I B	2010	Practitioner-supported delivery of internet-based cognitive behaviour therapy: evaluation of the feasibility of conducting a cluster randomised trial	Medical Journal of Australia
51	Hilvert-Bruce, Z	2012	Adherence as a determinant of effectiveness of internet cognitive behavioural therapy for anxiety and depressive disorders	Behaviour Research and Therapy
52	Hoek, W	2011	Randomized controlled trial of primary care physician motivational interviewing versus brief advice to engage adolescents with an Internet-based depression prevention intervention: 6-month outcomes and predictors of improvement	Translational Research: The Journal of Laboratory and Clinical Medicine
53	Hoek, W	2012	Effects of Internet-based guided self-help problem-solving therapy for adolescents with depression and anxiety: a randomized controlled trial	PLoS ONE
54	Hoifodt, R S	2013	The clinical effectiveness of web-based cognitive behavioral therapy with face-to-face therapist support for depressed primary care patients: randomized controlled trial	Journal of Medical Internet Research
55	Hollinghurst, S	2010	Cost-effectiveness of therapist-delivered online cognitive-behavioural therapy for depression: randomised controlled trial	British Journal of Psychiatry
56	Hunkeler, E M	2012	A web-delivered care management and patient self-management program for recurrent depression: a randomized trial	Psychiatric Services
57	Iloabachie, C	2011	Adolescent and parent experiences with a primary care/Internet-based depression prevention intervention (CATCH-IT)	General Hospital Psychiatry
58	Jacmon, John	2009	Treatment of major depression: Effectiveness of cognitive-behavioural therapy with an internet course as a central component	E-Journal of Applied Psychology
59	Johansson, R	2013	Choosing between Internet-based psychodynamic versus cognitive behavioral therapy for depression: a pilot preference study	BMC Psychiatry
60	Jones, R B	2012	Accuracy of geographically targeted internet advertisements on Google AdWords for recruitment in a randomized trial	Journal of Medical Internet Research
61	Jones, R B	2013	Recruitment to online therapies for depression: pilot cluster randomized controlled trial	Journal of Medical Internet Research
62	Kelders, S M	2013	Development of a web-based intervention for the indicated prevention of depression	BMC Medical Informatics and Decision Making
63	Kelders, S M	2013	Participants, usage, and use patterns of a web-based intervention for the prevention of	Journal of Medical Internet Research

			depression within a randomized controlled trial	
64	Kenicer, D	2012	A national survey of health service infrastructure and policy impacts on access to computerised CBT in Scotland	BMC Medical Informatics and Decision Making
65	Kenter, R	2013	Guided online treatment in routine mental health care: an observational study on uptake, drop-out and effects	BMC Psychiatry
66	Kerr, J	2008	A pilot study to assess the feasibility and acceptability of a community based physical activity intervention (involving Internet, telephone, and pedometer support), integrated with medication and mood management for depressed patients	Mental Health and Physical Activity
67	Kessler, D	2009	Therapist-delivered internet psychotherapy for depression in primary care: a randomised controlled trial	Lancet
68	Kojima, R	2010	Efficacy of Cognitive Behavioral Therapy Training Using Brief E-mail Sessions in the Workplace: A Controlled Clinical Trial	Industrial Health
69	Krusche, A	2013	Mindfulness online: an evaluation of the feasibility of a web-based mindfulness course for stress, anxiety and depression	BMJ Open
70	Kurki, M	2011	Usefulness of Internet in adolescent mental health outpatient care	Journal of Psychiatric and Mental Health Nursing
71	Kurki, M	2013	Integration of computer and Internet-based programmes into psychiatric out-patient care of adolescents with depression	Informatics for Health & Social Care
72	Landback, J	2009	From prototype to product: development of a primary care/Internet based depression prevention intervention for adolescents (CATCH-IT)	Community Mental Health Journal
73	Lemma, A	2013	Feasibility study of a psychodynamic online group intervention for depression	Psychoanalytic Psychology
74	Levin, M E	2014	Feasibility of a Prototype Web-Based Acceptance and Commitment Therapy Prevention Program for College Students	Journal of American College Health
75	Lillevoll, K R	2013	Patients' experiences of helpfulness in guided internet-based treatment for depression: qualitative study of integrated therapeutic dimensions	Journal of Medical Internet Research
76	Lillevoll, K R	2014	Uptake and adherence of a self-directed internet-based mental health intervention with tailored e-mail reminders in senior high schools in Norway	BMC Psychiatry
77	Lintvedt, O K	2008	The need for web-based cognitive behavior therapy among university students	Journal of Technology in Human Services
78	Lintvedt, O K	2013	Evaluating the effectiveness and efficacy of unguided internet-based self-help intervention for the prevention of depression: a randomized controlled trial	Clinical Psychology & Psychotherapy

79	Lintvedt, O K	2013	Evaluating the translation process of an Internet-based self-help intervention for prevention of depression: a cost-effectiveness analysis	Journal of Medical Internet Research
80	Lokkerbol, J	2014	Improving the cost-effectiveness of a healthcare system for depressive disorders by implementing telemedicine: a health economic modeling study	American Journal of Geriatric Psychiatry
81	Mailey, E L	2010	Internet-delivered physical activity intervention for college students with mental health disorders: a randomized pilot trial	Psychology, Health & Medicine
82	Mallen, M J	2011	Online counselling: An initial examination of the process in a synchronous chat environment	Counselling & Psychotherapy Research
83	Maloni, J A	2013	Web Recruitment and Internet Use and Preferences Reported by Women With Postpartum Depression After Pregnancy Complications	Archives of Psychiatric Nursing
84	Mansson, K N	2013	Development and initial evaluation of an internet-based support system for face-to-face cognitive behavior therapy: a proof of concept study	Journal of Medical Internet Research
85	Marko, M.	2010	Adolescent Internet depression prevention: Preferences for intervention and predictors of intentions and adherence	Journal of CyberTherapy and Rehabilitation
86	Mewton, L	2013	A naturalistic study of the acceptability and effectiveness of internet-delivered cognitive behavioural therapy for psychiatric disorders in older australians	PLoS ONE
87	Meyer, B	2009	Effectiveness of a novel integrative online treatment for depression (Deprexis): randomized controlled trial	Journal of Medical Internet Research
88	Meyer, D	2007	Online self-help: developing a student-focused website for depression... www.studentdepression.org	Counselling & Psychotherapy Research
89	Mohr, D C	2010	Interest in behavioral and psychological treatments delivered face-to-face, by telephone, and by internet	Annals of Behavioral Medicine
90	Mohr, D C	2010	Multimodal e-mental health treatment for depression: a feasibility trial	Journal of Medical Internet Research
91	Mongrain, M	2012	Do Positive Psychology Exercises Work? A Replication of Seligman et al. ()	Journal of Clinical Psychology
92	Mora, L	2008	Psychologist treatment recommendations for Internet-based therapeutic interventions	Computers in Human Behavior
93	Morgan, A J	2012	Email-based promotion of self-help for subthreshold depression: Mood Memos randomised controlled trial	British Journal of Psychiatry
94	Morgan, A J	2013	Behavior change through automated e-mails: Mediation analysis of self-help strategy use for depressive symptoms	Behaviour Research and Therapy
95	Morgan, A J	2013	Internet-based recruitment to a depression prevention intervention: lessons from the Mood Memos study	Journal of Medical Internet Research

96	Morgan, A J	2013	Self-help for depression via e-mail: A randomised controlled trial of effects on depression and self-help behaviour	PLoS ONE
97	Moritz, S	2012	A randomized controlled trial of internet-based therapy in depression	Behaviour Research and Therapy
98	Moritz, S	2013	The more it is needed, the less it is wanted: attitudes toward face-to-face intervention among depressed patients undergoing online treatment	Depression and Anxiety
99	Mota Pereira, J	2014	Facebook Enhances Antidepressant Pharmacotherapy Effects	Scientific World Journal
100	Naversnik, K	2013	Cost-effectiveness of a novel e-health depression service	Telemedicine Journal and e-Health
101	Neil, A L	2009	Predictors of adherence by adolescents to a cognitive behavior therapy website in school and community-based settings	Journal of Medical Internet Research
102	Newby, J M	2013	Internet cognitive behavioural therapy for mixed anxiety and depression: a randomized controlled trial and evidence of effectiveness in primary care	Psychological Medicine
103	Nordgreen, T	2011	Use of self-help materials for anxiety and depression in mental health services: A national survey of psychologists in Norway	Professional Psychology: Research and Practice
104	O'Kearney, R	2006	Effects of a cognitive-behavioural internet program on depression, vulnerability to depression and stigma in adolescent males: a school-based controlled trial	Cognitive Behaviour Therapy
105	O'Kearney, R	2009	A controlled trial of a school-based Internet program for reducing depressive symptoms in adolescent girls	Depression and Anxiety
106	O'Mahen, H A	2013	Internet-based behavioral activation-Treatment for postnatal depression (Netmums): A randomized controlled trial	Journal of Affective Disorders
107	O'Mahen, H A	2013	Netmums: a phase II randomized controlled trial of a guided Internet behavioural activation treatment for postpartum depression	Psychological Medicine
108	Patten, S B	2003	Prevention of depressive symptoms through the use of distance technologies	Psychiatric Services
109	Phillips, R	2014	Randomized controlled trial of computerized cognitive behavioural therapy for depressive symptoms: effectiveness and costs of a workplace intervention	Psychological Medicine
110	Pittaway, S	2009	Comparative, clinical feasibility study of three tools for delivery of cognitive behavioural therapy for mild to moderate depression and anxiety provided on a self-help basis	Mental Health in Family Medicine
111	Proudfoot, J	2013	Impact of a mobile phone and web program on symptom and functional outcomes for people with mild-to-moderate depression, anxiety and stress: a randomised controlled trial	BMC Psychiatry
112	Pugh, N E	2014	A Case Study Illustrating Therapist-Assisted Internet Cognitive Behavior Therapy for Depression	Cognitive and Behavioral Practice

113	Reynolds, D J	2013	Impact of Exchanges and Client-Therapist Alliance in Online-Text Psychotherapy	Cyberpsychology Behavior and Social Networking
114	Richards, D	2009	The experience of implementing, recruiting and screening for an online treatment for depression in a naturalistic setting	Counselling Psychology Review
115	Richards, D	2012	Client-identified helpful and hindering events in therapist-delivered vs. self-administered online cognitive-behavioural treatments for depression in college students	Counselling Psychology Quarterly
116	Richards, D	2013	A comparison of two online cognitive-behavioural interventions for symptoms of depression in a student population: The role of therapist responsiveness	Counselling & Psychotherapy Research
117	Richards, D	2013	Satisfaction with therapist-delivered vs. self-administered online cognitive behavioural treatments for depression symptoms in college students	British Journal of Guidance & Counselling
118	Robertson, L	2005	Case management and adherence to an online disease management system	Journal of Telemedicine and Telecare
119	Robertson, L	2006	Using the Internet to enhance the treatment of depression	Australasian Psychiatry
120	Rogers, V L	2009	Internet versus face-to-face therapy: emotional self-disclosure issues for young adults	Issues in Mental Health Nursing
121	Ruby, A.	2013	Economic Analysis of an Accountable Care Organization Centered Internet-Based Depression Prevention	The Journal of Mental Health Policy and Economics
122	Ruwaard, J	2009	Standardized web-based cognitive behavioural therapy of mild to moderate depression: a randomized controlled trial with a long-term follow-up	Cognitive Behaviour Therapy
123	Ruwaard, J	2012	The effectiveness of online cognitive behavioral treatment in routine clinical practice	PLoS ONE
124	Saulsberry, A	2013	Randomized Clinical Trial of a Primary Care Internet-based Intervention to Prevent Adolescent Depression: One-year Outcomes	Journal of the Canadian Academy of Child & Adolescent Psychiatry
125	Schneider, J	2014	Acceptability of online self-help to people with depression: users' views of MoodGYM versus informational websites.	Journal of Medical Internet Research
126	Schueller, S M	2012	Disseminating self-help: positive psychology exercises in an online trial	Journal of Medical Internet Research
127	Seligman, M E	2007	Group prevention of depression and anxiety symptoms	Behaviour Research and Therapy
128	Sergeant, S	2014	An Online Optimism Intervention Reduces Depression in Pessimistic Individuals	Journal of Consulting and Clinical Psychology Jan
129	Shapira, L B	2010	The benefits of self-compassion and optimism exercises for individuals vulnerable to depression	The Journal of Positive Psychology
130	Sharry, J	2013	A service-based evaluation of a therapist-supported online cognitive behavioral therapy program for depression	Journal of Medical Internet Research

131	Sheeber, L B	2012	Development and pilot evaluation of an Internet-facilitated cognitive-behavioral intervention for maternal depression	Journal of Consulting and Clinical Psychology
132	Silberman, J	2011	Reductions in Employee Productivity Impairment Observed After Implementation of Web-Based Worksite Health Promotion Programs	Journal of Occupational and Environmental Medicine
133	Simon, G E	2011	Randomized trial of depression follow-up care by online messaging	Journal of General Internal Medicine
134	Sobowale, K	2013	Adaptation of an internet-based depression prevention intervention for Chinese adolescents: From "CATCH-IT" to "grasp the opportunity"	International Journal of Adolescent Medicine and Health
135	Spek, V	2008	One-year follow-up results of a randomized controlled clinical trial on internet-based cognitive behavioural therapy for subthreshold depression in people over 50 years	Psychological Medicine
136	Spek, V	2008	Predictors of outcome of group and internet-based cognitive behavior therapy	Journal of Affective Disorders
137	Sunderland, M	2012	Investigating trajectories of change in psychological distress amongst patients with depression and generalised anxiety disorder treated with internet cognitive behavioural therapy	Behaviour Research and Therapy
138	Titov, N	2010	Characteristics of adults with anxiety or depression treated at an internet clinic: comparison with a national survey and an outpatient clinic	PLoS ONE
139	Titov, N	2010	Internet treatment for depression: a randomized controlled trial comparing clinician vs. technician assistance	PLoS ONE
140	Titov, N	2013	Improving adherence and clinical outcomes in self-guided internet treatment for anxiety and depression: randomised controlled trial	PLoS ONE
141	Titov, N	2014	Improving adherence and clinical outcomes in self-guided internet treatment for anxiety and depression: a 12-month follow-up of a randomised controlled trial	PLoS ONE
142	Unlu Ince, B	2013	Internet-based, culturally sensitive, problem-solving therapy for Turkish migrants with depression: randomized controlled trial	Journal of Medical Internet Research
143	Valimaki, M	2012	Developing an internet-based support system for adolescents with depression	JMIR Research Protocols
144	van der Zanden, R	2012	Effectiveness of an online group course for depression in adolescents and young adults: a randomized trial	Journal of Medical Internet Research
145	van der Zanden, R	2013	Online cognitive-based intervention for depression: exploring possible circularity in mechanisms of change	Psychological Medicine
146	Van Gemert-Pijnen, J E	2014	Understanding the usage of content in a mental health intervention for depression: an analysis of log data	Journal of Medical Internet Research
147	Van Voorhees, B W	2005	Pilot study of a primary care internet-based depression prevention intervention for late adolescents	The Canadian Child & Adolescent Psychiatry Review

148	Van Voorhees, B W	2007	Development and process evaluation of a primary care internet-based intervention to prevent depression in emerging adults	Primary Care Companion to the Journal of Clinical Psychiatry
149	Van Voorhees, B W	2008	Integrative internet-based depression prevention for adolescents: a randomized clinical trial in primary care for vulnerability and protective factors	Journal of the Canadian Academy of Child & Adolescent Psychiatry
150	Van Voorhees, B W	2009	Adolescent dose and ratings of an Internet-based depression prevention program: A randomized trial of primary care physician brief advice versus a motivational interview	Journal of Cognitive and Behavioral Psychotherapies
151	Van Voorhees, B W	2009	Randomized clinical trial of an Internet-based depression prevention program for adolescents (Project CATCH-IT) in primary care: 12-week outcomes	Journal of Developmental and Behavioral Pediatrics
152	Van Voorhees, B W	2010	Adolescents in primary care with sub-threshold depressed mood screened for participation in a depression prevention study: Co-morbidity and factors associated with depressive symptoms	The Open Psychiatry Journal
153	Van Voorhees, B W	2010	Development and pilot study of a marketing strategy for primary care/internet-based depression prevention intervention for adolescents (the CATCH-IT intervention)	Primary Care Companion to the Journal of Clinical Psychiatry
154	Vangberg, H C	2012	Does Personality Predict Depression and Use of an Internet-Based Intervention for Depression among Adolescents?	Depression Research and Treatment
155	Warmerdam, L	2010	Cost-utility and cost-effectiveness of internet-based treatment for adults with depressive symptoms: randomized trial (Provisional abstract)	Journal of Medical Internet Research
156	Watkins, D C	2011	Email reminders as a self-management tool in depression: a needs assessment to determine patients' interests and preferences	Journal of Telemedicine and Telecare
157	Watts, S	2012	A clinical audit of changes in suicide ideas with internet treatment for depression	BMJ Open
158	Watts, S	2013	CBT for depression: A pilot RCT comparing mobile phone vs. computer	BMC Psychiatry
159	Wilhelmsen, M	2013	Motivation to persist with internet-based cognitive behavioural treatment using blended care: a qualitative study	BMC Psychiatry
160	Williams, A D	2013	Combining imagination and reason in the treatment of depression: a randomized controlled trial of internet-based cognitive-bias modification and internet-CBT for depression	Journal of Consulting and Clinical Psychology
161	Williams, A D	2013	The effectiveness of Internet cognitive behavioural therapy (iCBT) for depression in primary care: a quality assurance study	PLoS ONE
162	Williams, A D	2013	The impact of psychological distress tolerance in the treatment of depression	Behaviour Research and Therapy
163	Wojtowicz, M	2013	Predictors of participant retention in a guided online self-help program for university students: prospective cohort study	Journal of Medical Internet Research

164	Woodford, J	2011	Recruitment into a guided internet based CBT (iCBT) intervention for depression: lesson learnt from the failure of a prevalence recruitment strategy	Contemporary Clinical Trials
